# Genetic risk, incident stroke, and the benefits of adhering to a healthy lifestyle: cohort study of 306 473 UK Biobank participants

**DOI:** 10.1136/bmj.k4168

**Published:** 2018-10-24

**Authors:** Susanna C Larsson, Rainer Malik, Kristiina Rannikmäe, MEGASTROKE consortium, International Stroke Genetics Consortium, Cathie L Sudlow, Martin Dichgans, Hugh S Markus, Matthew Traylor, Loes CA Rutten-Jacobs

**Affiliations:** 1 German Center for Neurodegenerative diseases (DZNE), Population Health Sciences, Sigmund-Freud-Strasse 27, 53127 Bonn, Germany; 2 Department of Clinical Neurosciences, Stroke Research Group, University of Cambridge, UK; 3 Unit of Nutritional Epidemiology, Institute of Environmental Medicine, Karolinska Institutet, Stockholm, Sweden; 4 Institute for Stroke and Dementia Research (ISD), University Hospital, LMU Munich, Munich, Germany; 5 Centre for Medical Informatics, Usher Institute of Population Health Sciences and Informatics, University of Edinburgh, Edinburgh, UK; 6 Centre for Clinical Brain Sciences, University of Edinburgh, Edinburgh, UK; 7 Institute of Genetics and Molecular Medicine, University of Edinburgh, Edinburgh, UK; 8 Munich Cluster for Systems Neurology (SyNergy), Munich, Germany; 9 German Center for Neurodegenerative Diseases (DZNE), Munich, Germany

## Abstract

**Objective:**

To evaluate the associations of a polygenic risk score and healthy lifestyle with incident stroke.

**Design:**

Prospective population based cohort study.

**Setting:**

UK Biobank Study, UK.

**Participants:**

306 473 men and women, aged 40-73 years, recruited between 2006 and 2010.

**Main outcome measure:**

Hazard ratios for a first stroke, estimated using Cox regression. A polygenic risk score of 90 single nucleotide polymorphisms previously associated with stroke was constructed at P<1×10^−5^ to test for an association with incident stroke. Adherence to a healthy lifestyle was determined on the basis of four factors: non-smoker, healthy diet, body mass index <30 kg/m^2^, and regular physical exercise.

**Results:**

During a median follow-up of 7.1 years (2 138 443 person years), 2077 incident strokes (1541 ischaemic stroke, 287 intracerebral haemorrhage, and 249 subarachnoid haemorrhage) were ascertained. The risk of incident stroke was 35% higher among those at high genetic risk (top third of polygenic score) compared with those at low genetic risk (bottom third): hazard ratio 1.35 (95% confidence interval 1.21 to 1.50), P=3.9×10^−8^. Unfavourable lifestyle (0 or 1 healthy lifestyle factors) was associated with a 66% increased risk of stroke compared with a favourable lifestyle (3 or 4 healthy lifestyle factors): 1.66 (1.45 to 1.89), P=1.19×10^−13^. The association with lifestyle was independent of genetic risk stratums.

**Conclusion:**

In this cohort study, genetic and lifestyle factors were independently associated with incident stroke. These results emphasise the benefit of entire populations adhering to a healthy lifestyle, independent of genetic risk.

## Introduction

Stroke is one of the leading reasons for disability and death worldwide.^1^ It is a complex disease, caused by both genetic and environmental factors, including diet and lifestyle.^2^

Early evidence supporting a role for genetics in risk of stroke came from twin studies and family history studies.^3^ ^4^ Further evidence has emerged from genome wide association studies. MEGASTROKE, a large meta-analysis of genome wide association studies tripled the number of loci robustly associated with stroke risk.^5^

Lifestyle is an important modifiable risk factor for stroke. Clear evidence shows that adhering to a healthy lifestyle, including not smoking, reducing the risk of diabetes, regular physical activity, and a healthy diet, decreases the risk of stroke substantially.^6^ ^7^ It might be hypothesised that adhering to a healthy lifestyle could attenuate the effect of genetics on stroke risk. A previous study in coronary artery disease—a condition closely related to ischaemic stroke, found a statistically significant interplay between genetic and lifestyle risk factors in the risk of coronary artery disease.^8^

We investigated whether a weighted genetic risk score based on the genome wide association results for stroke in MEGASTROKE is associated with incident stroke in a large population based cohort (UK Biobank). We also investigated whether adherence to a healthy lifestyle influences this association.

## Methods

### Study population

UK Biobank is a prospective study that recruited 500 000 community dwelling participants, aged 40-69 years, from across the United Kingdom between 2006 and 2010 (www.ukbiobank.ac.uk).^9^ Participants attended one of 22 assessment centres across England, Scotland, and Wales. The study collects extensive data from questionnaires, interviews, health records, physical measures, biological samples, and imaging. Main outcomes and exposures of interest in the current study include imputed genetic data, incident stroke, and lifestyle (smoking, diet, body mass index, and physical activity).

In the present study we included all people who were classified as white British (all who self identified as white British, followed by the exclusion of ethnic outliers identified by principal components analysis on the genotype data), without a history of stroke or myocardial infarction on the basis of self report or medical records, or both, and with complete data on lifestyle.

### Healthy lifestyle factors

We defined four healthy lifestyle factors on the basis of the American Heart Association guidelines: no current smoking, healthy diet, body mass index <30 kg/m^2^, and moderate physical activity two or more times weekly.^10^

The UK Biobank participants completed a questionnaire on their usual diet pattern. In this analysis, a healthy diet was determined according to the increased consumption of fruit, vegetables, and fish and the decreased consumption of processed meats and red meats. We defined a healthy diet as adherence to at least two of the healthy food items. Supplemental text S1 and table S1 provide additional details on the specific questions asked and the construction of a healthy diet score.

Moderate physical activity was defined as at least 150 minutes of moderate intensity activity weekly or 75 minutes of vigorous activity weekly.

### Incident stroke

Incident stroke in UK Biobank was based on medical history and linkage to data on hospital admissions and mortality. We used the stroke variables provided by UK Biobank, which were created by combining information from these different data sources (see supplemental table S2). Details of the algorithms used to combine the data from different sources to identify stroke have been described previously and are available on the UK Biobank website (www.ukbiobank.ac.uk). We subtyped stroke as ischaemic stroke, intracerebral haemorrhage, or subarachnoid haemorrhage.

We took into account the occurrence of myocardial infarction during the study as this could potentially result in lifestyle changes during follow-up that affect the risk of stroke. The occurrence of myocardial infarction was defined according to the UK Biobank algorithmic definition (see supplemental table S2). Details of the myocardial infarction algorithm have been described previously and are available on the UK Biobank website (www.ukbiobank.ac.uk).^11^

We excluded people from the analysis who self reported stroke or myocardial infarction.

### Genetic data

We used the June 2017 release of the imputed genetic data from UK Biobank (downloaded 3 June 2017). Details of the design of the arrays, sample processing, and stringent quality control have been described in detail elsewhere^12^ and summarised previously.^13^ Briefly, we used two closely related arrays from Affymetrix, the UK BiLEVE Axiom array (9.9% of people) and the UK Biobank Axiom array, to genotype about 805 426 markers with good genome wide coverage. Phasing was performed using SHAPEIT3 and imputation using IMPUTE4.^12^ ^15^ Two reference panels were used for imputation; the Haplotype Reference Consortium reference panel (39 131 578 autosomal single nucleotide polymorphisms, SNPs) and a merged UK10K and 1000 Genomes Phase 3 panel.^14^ Imputed genotypes were available for 488 369 participants in this study.^12^ From the resulting dataset, we excluded those who self reported ancestry other than white British, related people (second degree or greater: kinship coefficient ≥0.884), people with high levels of heterozygosity and missingness (>5%), and people whose reported sex was inconsistent with sex inferred from the genetic data. The UK Biobank core team centrally performed a check for excessive heterozygosity.^13^ Extreme heterozygosity or high rates of missingness, or both, can be indicators of poor sample quality due to, for example, DNA contamination. UK Biobank provided a list of samples with unusually high heterozygosity and we excluded those samples according to its recommendations. To evaluate a mismatch in sex self reported sex was compared with sex inferred from the genetic data (based on relative intensity of markers on the Y and X chromosomes). This sex mismatch evaluation was centrally performed by the UK Biobank core team and is described in detail elsewhere.^12^ This evaluation can be used as a way to detect sample mishandling or other kinds of clerical error. However, in a dataset of this size, some such mismatches would be expected owing to transgender people or instances of real (but rare) genetic variation, such as aneuploidies in sex chromosomes.

In this analysis we only included SNPs imputed from the Haplotype Reference Consortium panel.

### Polygenic risk score derived from MEGASTROKE

We derived three sets of independent (r^2^<0.05 or 1000 Kb apart) SNPs associated with any stroke in people of European ancestry in MEGASTROKE (see supplemental text S2) at P<5×10^−8^, P<1×10^−6^, and P<1×10^−5^ using an LD clumping procedure employed using plink v1.90b3.45.^5^ ^16^ For each individual in the UK Biobank sample we calculated quantitative aggregate risk scores, defined as the sum of the number of risk alleles present at each locus weighted by the log of the odds ratio for that locus estimated from the MEGASTROKE sample using the plink “–score” command.

The three polygenic scores were tested for an association with incident any stroke, and for further analyses we used the polygenic risk score most statistically significantly associated with incident stroke. Supplemental table S3 lists the SNPs included in the MEGASTROKE risk score (all stroke, P<1×10^−5^).

We repeated the previous steps while restricting to ischaemic stroke in those of European ancestry to create a genetic risk score for ischaemic stroke (P<1×10^−5^).

### Statistical analysis

We defined genetic risk in thirds: “low risk” (lowest third of genetic risk score), “intermediate risk” (second third), “high risk” (highest third). Lifestyle was recorded as “favourable” (three or four healthy lifestyle factors), “intermediate” (two healthy lifestyle factors), “unfavourable” (no or one healthy lifestyle factor).

To test the association of genetic and lifestyle factors with incident stroke we used Cox proportional hazards models. The duration of follow-up was calculated as time between the baseline assessment and the first event of either stroke, myocardial infarction, death, or 1 March 2016, which was the end of follow-up for the current data release. Participants who had a myocardial infarction or died before a stroke occurred were censored at the time of the respective event.

We repeated the previous steps for the ischaemic stroke genetic risk score and assessed the association between this score and incident ischaemic stroke. In this analysis we censored participants with a diagnosis of intracerebral haemorrhage, subarachnoid haemorrhage, or myocardial infarction or who died before an ischaemic stroke occurred at the time of the respective event.

Cox proportional hazards models included adjustment for age and sex for the lifestyle score models. For the models including the genetic score we additionally adjusted for the first 10 principal components of ancestry and genotyping batch. Model discrimination was evaluated with the concordance (*c*) statistic.

We included an interaction term in the regression model to test for statistical interaction between the lifestyle and genetic risk score.

To obtain cumulative incidence for lifestyle and genetic risk stratums we used competing risk regression; the cumulative incidence function. We compared the hazard ratios for the genetic and lifestyle score in the risk of stroke derived from Cox proportional hazards models with the subdistribution hazard ratios, calculated using proportional subdistribution hazards regression models.^17^

R software version 3.4.2 was used for the Cox proportional hazards regression (package “survival”) and proportional subdistribution hazards regression (package “cmprsk”).

### Patient and public involvement

The development of the research question or outcome measures was not informed by patients’ priorities, experience, or preferences. No patients were involved in the design and conduct of the present study. There are no plans to disseminate the results to study participants.

## Results

**Fig 1:**
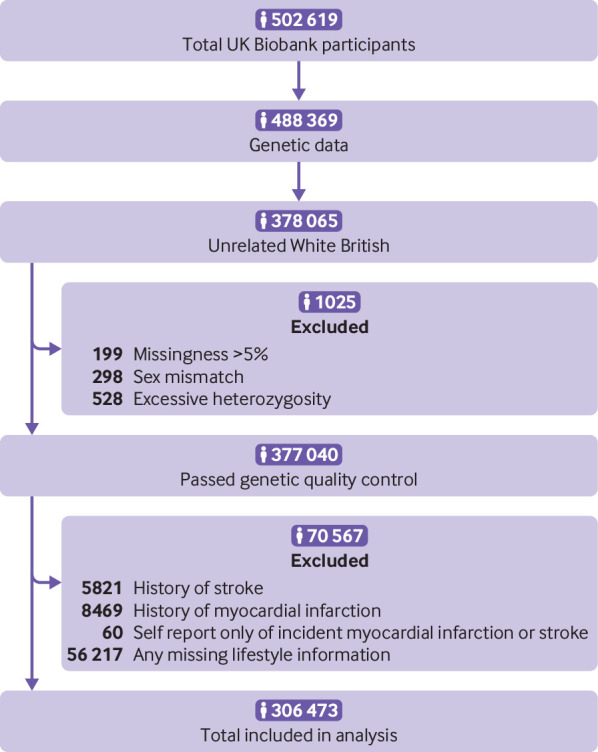
Flow of participants through study

Complete data for the present analysis were available for 306 473 participants in the UK Biobank Study (fig 1). Table 1 shows the baseline characteristics of the study population. During a total of 2 138 443 person years (median follow-up 7.1 years), 2077 incident fatal or non-fatal strokes were reported as first incident vascular event or death, of which 1541 were ischaemic stroke, 287 intracerebral haemorrhage, and 249 subarachnoid haemorrhage. Furthermore, 3436 cases of fatal or non-fatal myocardial infarction and 6646 deaths due to other causes than stroke or myocardial infarction were reported as first incident vascular event or death.

**Table 1:** Characteristics of participants at baseline. Values are numbers (participants) unless stated otherwise

Characteristic	All (n=306 473)	Incident stroke (n=2077)	No stroke (n=304 396)
Mean (SD) age (years)	56.7 (7.9)	61.2 (6.8)	56.6 (7.9)
Men	136 654 (44.6)	1184 (57.0)	135 470 (44.5)
Mean (SD) systolic blood pressure (mm Hg)	138 (18)	146 (21)	138 (19)
Mean (SD) diastolic blood pressure (mm Hg)	82 (10)	85 (11)	82 (10)
Mean (SD) body mass index	27.2 (4.7)	28.0 (4.8)	27.2 (4.7)
Diabetes	12 927 (4.2)	209 (10.1)	1865 (4.2)
Use of lipid lowering drugs	44 785 (14.7)	497 (24.1)	44 288 (14.6)
Use of antihypertensives	61 218 (20.0)	697 (33.6)	60 521 (19.9)
Healthy lifestyle factors:			
No current smoking	286 352 (93.4)	1822 (87.7)	284 530 (93.5)
Body mass index <30	236 326 (77.1)	1489 (71.7)	588 (77.1)
Regular moderate physical activity	181 234 (59.1)	1203 (57.9)	180 031 (59.1)
Healthy diet	139 328 (45.5)	884 (42.6)	138 444 (45.5)
Healthy lifestyle score:			
Favourable (3 or 4 healthy lifestyle factors)	191 003 (62.3)	1157 (55.7)	189 846 (62.4)
Intermediate (2 healthy lifestyle factors)	86 710 (28.3)	652 (31.4)	86 058 (28.3)
Unfavourable (0 or 1 healthy lifestyle factor)	28 760 (9.4)	268 (12.9)	28 492 (9.4)
Genetic risk category:			
Low	101 977 (33.3)	589 (28.4)	101 388 (33.3)
Intermediate	102 300 (33.4)	703 (33.8)	101 597 (33.4)
High	102 196 (33.3)	785 (37.8)	101 411 (33.3)

Polygenic risk scores containing independent SNPs (on basis of linkage disequilibrium patterns) derived from MEGASTROKE at three different significance thresholds were tested for association with incident stroke in UK Biobank (see supplemental figure S1). The three polygenic risk scores were associated with risk of incident stroke, but the genetic risk score including all SNPs associated with stroke in MEGASTROKE at P<1×10^−5^ (90 SNPs, see supplemental table S3) showed the strongest association and was therefore selected for subsequent analyses. The polygenic risk approximated a normal distribution (see supplemental figure S2).

**Fig 2:**
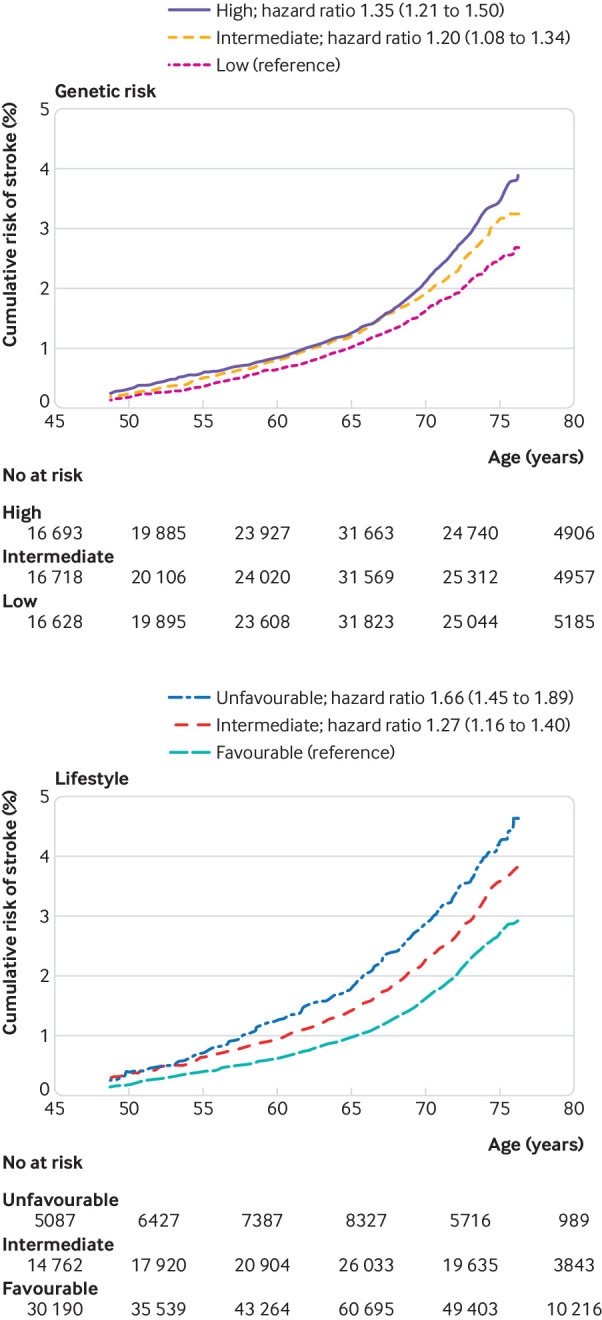
Standardised risk of incident stroke according to genetic risk and lifestyle profile. Cox proportional hazards models were adjusted for age and sex, and the genetic risk models included additionally the first 10 principal components of ancestry and genotyping batch

In Cox proportional hazards analysis, the risk of incident stroke was higher for those with intermediate (hazard ratio 1.20, 95% confidence interval 1.08 to 1.34) and high genetic risk scores (1.35, 1.21 to 1.50) compared with those with a low genetic risk score (fig 2). We tested available cardiometabolic risk factors for an association with the genetic risk score, adjusting for the first 10 principal components of ancestry, genotyping batch, age, and sex. The genetic risk score was significantly associated with systolic blood pressure (P=1.5×10^−15^), diastolic blood pressure (P=1.1×10^−7^), use of lipid lowering drugs (P=7.5×10^−13^), and diabetes (7.6×10^−4^), but not with body mass index (P=0.18).

The cumulative risk of stroke increased with age. Blood pressure, diabetes, and the use of lipid lowering drugs did not seem to influence the association between genetic risk of stroke and incident stroke (see supplemental table S4). Similarly, the risk of stroke was increased in those with an unfavourable (hazard ratio 1.66, 95% confidence interval 1.45 to 1.89) and intermediate (1.27, 1.16 to 1.40) lifestyle compared with those with a favourable lifestyle (fig 2).

Supplemental figure S3 shows the distribution of thirds of genetic risk and lifestyle scores. The genetic risk score was not associated with any of the single healthy lifestyle factors: odds ratio 0.98 (95% confidence interval 0.93 to 1.04) for body mass index score, 1.04 (0.99 to 1.09) for diet score, 0.97 (0.88 to 1.07) for smoking score, and 0.98 (0.94 to 1.03) for exercise score. Furthermore, the association of genetic risk with incident stroke was unchanged after adjustment for lifestyle (see supplemental table S5). Likewise, the association of lifestyle with incident stroke was essentially unchanged after adjustment for the genetic risk score (see supplemental table S5).

Table 2 shows the risk of incident stroke for combined genetic risk and lifestyle profiles. An additive effect was found for genetic risk and lifestyle on risk of incident stroke. Within each genetic risk stratum there was an increase in strength of association with decreasing number of favourable life style factors (see supplemental table S6). The highest risk of incident stroke was observed in participants with a high genetic risk and an unfavourable lifestyle: hazard ratio 2.30 (95% confidence interval 1.84 to 2.87); see supplemental figure S4. The test for statistical interaction between lifestyle score and genetic risk score in relation to incident stroke was not significant (P=0.57) compared with participants with low genetic risk and favourable lifestyle.

**Table 2:** Relative and absolute risk of incident stroke according to genetic and lifestyle profiles

Genetic risk	Lifestyle		
	Favourable	Intermediate	Unfavourable
**Low**			
Hazard ratio* (95% CI)	1 (reference)	1.36 (1.14 to 1.63), P=7.3×10^−04^	1.84 (1.44 to 2.35), P=8.0×10^−07^
8 year cumulative incidence† (%) (95% CI)	0.54 (0.47 to 0.60)	0.74 (0.63 to 0.85)	0.95 (0.74 to 1.17)
**Intermediate**			
Hazard ratio* (95% CI)	1.26 (1.09 to 1.46), P=0.002	1.62 (1.37 to 1.92), P=3.2×10^−08^	1.85 (1.46 to 2.37), P=5.4×10^−07^
8 year cumulative incidence† (%) (95% CI)	0.67 (0.60 to 0.74)	0.82 (0.71 to 0.93)	0.92 (0.72 to 1.12)
**High**			
Hazard ratio* (95% CI)	1.44 (1.25 to 1.66), P=7.0×10^−07^	1.70 (1.44 to 2.01), P=8.1×10^−10^	2.30 (1.84 to 2.87), P=3.3×10^−13^
8 year cumulative incidence† (%) (95% CI)	0.78 (0.70 to 0.86)	0.91 (0.78 to 1.04)	1.11 (0.89 to 1.33)

* Calculated using Cox proportional hazards models, adjusted for age, sex, first 10 principal components of ancestry, and genotyping batch.

† Calculated using the cumulative incidence function as implemented in the “cmprsk” R package.

Model discrimination was similar between the main Cox proportional hazards models including the genetic risk score (*c* statistic 0.69 (SE) 0.01), lifestyle score (0.69 (SE 0.01)), and the combined genetic and lifestyle score (0.70 (SE 0.01)).

We repeated the analysis to test the associations of the genetic and lifestyle scores with incident stroke while restricting only to ischaemic stroke for both genetic risk score and outcome. In addition, we compared the results derived from the Cox proportional hazards model with those derived from the competing risk proportional subdistribution hazards model. Results did not change substantially when restricting to ischaemic stroke or when using the subdistribution hazards model compared with the original analyses (see supplementary tables S7-S9).

Among individual components of the lifestyle score, smoking and body mass index ≥30 kg/m^2^ contributed most to the risk of incident stroke (table 3). For all lifestyle factors, the effects were similar across genetic risk stratums.

**Table 3:** Multivariable Cox regression analysis of age, sex, and lifestyle factors in relation to risk of stroke, stratified by genetic risk

Lifestyle factors	Low genetic risk			Moderate genetic risk			High genetic risk	
	Hazard ratio (95% CI)	P value		Hazard ratio (95% CI)	P value		Hazard ratio (95% CI)	P value
Age, per year	1.08 (1.07 to 1.09)	<2×10^−16^		1.09 (1.08 to 1.10)	<2×10^−16^		1.11 (1.10 to 1.12)	<2×10^−16^
Male sex	1.47 (1.25 to 1.74)	4.6×10^−06^		1.65 (1.42 to 1.93)	1.2×10^−10^		1.56 (1.35 to 1.80)	1.4×10^−09^
Current smoking	2.35 (1.84 to 3.01)	7.3×10^−12^		2.81 (2.27 to 3.48)	<2×10^−16^		1.87 (1.48 to 2.37)	1.5×10^−07^
Body mass index ≥30	1.43 (1.20 to 1.71)	8.0×10^−05^		1.38 (1.17 to 1.63)	1.2×10^−04^		1.19 (1.02 to 1.40)	0.03
No regular moderate physical activity	1.09 (0.92 to 1.28)	0.32		0.99 (0.85 to 1.63)	0.91		1.09 (0.94 to 1.26)	0.24
Unhealthy diet	1.10 (0.93 to 1.30)	0.27		1.11 (0.77 to 1.05)	0.16		1.21 (1.05 to 1.40)	0.01

As the effect of smoking was about twice as strong as the other individual lifestyle scores (table 3). We repeated the analysis of the risk of incident stroke for combined genetic risk and lifestyle profile in which smoking was counted twice, and this resulted in slightly increased point estimates in the unfavourable versus favourable lifestyle categories (see supplementary table S10).

The associations of genetic risk score with incident stroke were consistent in men and women (interaction P=0.70, supplemental figure S5). However, across all genetic risk stratums the absolute risk of incident stroke was lower in women than in men.

A statistically significant interaction on the multiplicative scale was found between sex and lifestyle profile in the risk of incident stroke (interaction P=0.01, supplemental figure S6). For men there was an increase in association with decreasing number of healthy lifestyle factors: hazard ratio 1.20 (95% confidence interval 1.05 to 1.36) and 1.82 (1.55 to 2.15) for intermediate and unfavourable lifestyle versus favourable lifestyle, respectively. However, among women there was no difference between intermediate and unfavourable lifestyle versus favourable lifestyle: 1.39 (1.21 to 1.61) and 1.36 (1.08 to 1.72), respectively.

## Discussion

We investigated the association between genetic risk of stroke, lifestyle, and incident risk of stroke in 306 473 people within the population based UK Biobank study. Risk of incident stroke was 35% higher among those at high genetic risk compared with those at low genetic risk, and these associations were independent of lifestyle profile. Furthermore, an unfavourable lifestyle was associated with a 66% increased risk of incident stroke compared with a favourable lifestyle, and this increased risk was present within any genetic risk category. A high genetic risk combined with an unfavourable lifestyle profile was associated with a more than twofold increased risk of stroke compared with a low genetic risk and a favourable lifestyle.

The present study provides further support that common genetic variants are implicated in the development of stroke. Our findings showing that a polygenic risk score is associated with incident stroke is in line with both clinical and population based studies.^18^ ^19^ ^20^ ^21^

The genetic risk score was also associated with blood pressure and use of lipid lowering drugs, which suggest that the effect of the genetic variants on risk of incident stroke might at least in part be mediated by vascular risk factors. However, adjusting for those factors did not change the effect size of association between genetic risk and incident stroke, which emphasises that other mechanisms than those that involve the traditional cardiovascular risk factors are likely important. In the MEGASTROKE genome wide association analysis of stroke, only about half of the identified loci shared genetic variation with related vascular traits, including blood pressure and lipid levels, which support that the genetic risk might act through additional mechanisms.^5^

The reduction of stroke risk by adhering to a healthy lifestyle has been well reported.^7^ ^22^ ^23^ ^24^ ^25^ The risk reduction associated with adherence to a healthy lifestyle in the present study was similar across all stratums of genetic risk, which emphasises the benefit for entire populations of adhering to a healthy lifestyle, independent of genetic risk. Among the lifestyle factors, the most statistically significant associations were observed for smoking and body mass index ≥30 kg/m^2^.

### Comparison with previous studies

Across all categories of genetic risk and lifestyle, the risk of incident stroke was higher in men than women. This is an expected finding given the previously consistently shown higher incidence of stroke in men compared with women at the age of most of the UK Biobank participants.^26^ Our results suggested that the relative risk of incident stroke associated with high genetic risk versus low genetic risk was similar in men and women. A family history and genome wide association studies suggested that genetic susceptibility to stroke is somewhat stronger in women than in men.^27^ ^28^ The methodological differences of those previous studies and the current study might explain the different conclusions. In the current study, only genetic variants associated with stroke in MEGASTROKE at P<1×10^−5^ were considered, whereas the other studies evaluated all genome wide variants within the study population or family history, which also includes environmental effects.

Considerable evidence from previous epidemiological studies also suggests differences in risk factors that are associated with stroke in men compared with women. Women have a higher prevalence of hypertension, whereas men have a higher prevalence of heart disease, diabetes, and unhealthy lifestyle behaviours, including smoking, obesity, and alcohol use.^29^ ^30^ ^31^ In the present study we found a higher relative risk of stroke associated with an unfavourable lifestyle in men than women (82% *v* 36% increased risk, respectively).

Other possibilities for the increased relative risk include potential differences in duration of exposure to unfavourable lifestyle factors. Future studies are needed to evaluate the effect of the duration of exposure to an unfavourable lifestyle profile on the risk of stroke.

### Strengths and limitations of this study

The major strengths of the current study include the large sample size of UK Biobank participants, which enabled study of the combination of genetic risk and lifestyle in detail. Furthermore, to derive a genetic risk score for stroke, we used MEGASTROKE, which is currently the largest genome wide association study of stroke.^5^ Another distinctive feature of this analysis compared with a previous study is that we also included single nucleotide polymorphisms (SNPs) associated with stroke at a subthreshold level of significance (P<1×10^−5^).^19^ This concurs with a previous study examining the predictive utility of genetic risk scores for incident coronary heart disease, which showed that the best performance was achieved by including SNPs that did not necessarily reach the genome wide statistical significance threshold in previous genome wide association studies.^32^

Our study has several limitations. Firstly, behavioural changes before or after the baseline examinations might have had an effect on the risk estimates. We tried to reduce the effect of behavioural changes that could be related to vascular disease by excluding all those with a history of stroke and by censoring those at the time a myocardial infarction occurred. Secondly, this analysis focused on a narrow range of lifestyle factors, based on the American Heart Association guidelines.^10^ Expanding the range of lifestyle factors (ie, stress, sleep, alcohol and drug use) and more detailed assessment of diet and physical activity would be of interest for future studies. Thirdly, in the current study we only evaluated stroke of any cause. The effects of lifestyle and genetic variants might differ according to the cause of stroke, although some genetic risk variants and vascular risk factors are shared between two or more causal factors.^5^

Finally, in the present study we restricted our analysis to people of European descent. Therefore, our results may not be generalisable to populations with distinct ancestry. Future studies are needed that test these relations in more diverse populations.

### Conclusion

In the present prospective population based cohort study of 306 473 people we found that genetic and lifestyle factors were independently associated with risk of incident stroke. These findings highlight the potential of lifestyle interventions to reduce risk of stroke across entire populations, even in those at high genetic risk of stroke.

**What is already known on this topic**

Stroke is a complex disease caused by both genetic and environmental factors, including diet and lifestyle
Whether adhering to a healthy lifestyle could attenuate the effect of genetic background on risk of incident stroke is currently unknown

**What this study adds**

Genetic and lifestyle factors were independently associated with risk of incident stroke
An unfavourable lifestyle profile was associated with increased risk of stroke across all genetic risk stratums
These findings highlight the potential of lifestyle interventions to reduce risk of stroke across entire populations, even in those at high genetic risk of stroke

## Web extra

Extra material as supplied by authors

**Supplementary information:** additional material

**Supplementary table: **table S3 showing SNPs included in MEGASTROKE risk score

## Notes

Contributors: LCAR-J and MT conceived the study. LCAR-J and MT wrote the first and successive drafts of the manuscript. LCAR-J and MT analysed the data. SCL contributed to study conception and design. All authors revised the manuscript for important intellectual content. LCAR-J and MT had full access to the data and take responsibility for the integrity of the data and the accuracy of the data analysis. LCAR-J is the guarantor. The corresponding author attests that all listed authors meet authorship criteria and that no others meeting the criteria have been omitted.

Ethical approval: UK Biobank received ethical approval from the research ethics committee (reference 13/NW/0382). All participants provided informed consent to participate. The present analyses were conducted under UK Biobank application number 19463.

Data sharing: The genetic and phenotypic UK Biobank data are available on application to the UK Biobank (www.ukbiobank.ac.uk/). Summary statistics from the MEGASTROKE meta-analysis of genome wide association studies in stroke and stroke subtypes are available from www.megastroke.org.

Transparency: The lead author (LCAR-J) affirms that the manuscript is an honest, accurate, and transparent account of the study being reported; that no important aspects of the study have been omitted; that discrepancies from the study as planned have been explained, and that the paper conforms to transparency policy of the International Committee of Medical Journal Editors uniform requirement for manuscripts submitted to biomedical journals.
