# Supplementary material for: Genetic risk, incident stroke, and the benefits of adhering to a healthy lifestyle: cohort study of 306 473 UK Biobank participants
Source: BMJ. 2018 Oct 24;363:k4168. doi: 10.1136/bmj.k4168 (PMC6199557; doi:10.1136/bmj.k4168)
Supplement: Supplementary file 2 — Supplementary table: table S3 showing SNPs included in MEGASTROKE risk score [file rutl044985.wt1.pdf]

SNP_ID	RISK_ALLELE	BETA	PVALUE
rs1052053	A	0.0675	2.25E-12
rs1070252	T	0.0662	1.77E-06
rs10774624	A	-0.0654	4.04E-12
rs10883926	A	0.0503	8.15E-08
rs10937513	A	0.0989	5.02E-07
rs11045896	A	0.0487	7.44E-06
rs11066283	A	-0.0692	2.36E-11
rs11242678	T	0.0643	8.71E-10
rs114591768	T	0.3355	2.43E-06
rs117426542	A	0.1487	1.56E-06
rs11952498	T	0.0431	5.69E-06
rs11957829	A	0.0616	1.51E-06
rs12326831	A	-0.0857	7.93E-06
rs12361415	T	0.0458	9.53E-06
rs12445022	A	0.052	1.03E-07
rs12562305	A	0.0882	2.60E-06
rs12630390	A	0.1184	3.72E-06
rs13312215	A	-0.1103	9.26E-06
rs143268049	T	-0.191	2.65E-07
rs147076266	A	-0.2051	3.56E-06
rs147410905	A	-0.1325	1.89E-06
rs147861947	A	0.1342	4.51E-06
rs148159238	T	0.1486	8.86E-06
rs149520112	T	0.1794	8.79E-06
rs1495900	A	-0.0479	5.97E-06
rs151080514	T	0.2419	5.58E-06
rs1537375	T	-0.0519	1.24E-08
rs1537407	T	-0.0662	4.48E-07
rs1549758	T	0.0532	3.11E-07
rs1563788	T	0.0449	6.14E-06
rs159963	A	0.0424	8.43E-06
rs17035646	A	0.0522	6.12E-08
rs187555384	A	0.0567	9.09E-06
rs2060213	T	-0.0523	1.28E-06
rs2066864	A	0.0562	1.29E-07
rs2107595	A	0.0803	3.59E-11
rs2284665	T	-0.0602	5.99E-08
rs2455134	A	-0.0564	8.58E-06
rs2585193	A	0.0504	2.45E-07
rs2634074	A	-0.084	6.56E-14
rs2978551	T	0.0446	3.39E-06
rs34212978	T	-0.0687	5.79E-06
rs34416434	T	-0.1155	1.39E-06
rs35046086	A	0.2006	7.19E-06
rs35276016	T	0.0984	9.31E-08

rs36053597	T	0.061	4.55E-07
rs4140660	T	0.0641	5.34E-06
rs4151702	C	-0.0551	2.63E-06
rs42039	T	-0.0574	1.65E-07
rs4556502	A	0.1383	1.23E-06
rs475937	A	0.0757	2.92E-08
rs4886564	T	-0.0653	2.37E-06
rs4903725	T	0.0586	9.27E-06
rs4942561	T	0.064	2.05E-09
rs57269940	T	0.0499	3.85E-06
rs59844791	A	-0.0633	8.15E-06
rs635634	T	0.0685	6.03E-08
rs650724	A	-0.0793	2.17E-06
rs6544653	T	-0.0451	8.84E-06
rs6838973	T	-0.0421	6.48E-06
rs6872625	T	0.051	4.21E-06
rs7124178	T	-0.0524	8.98E-07
rs7200604	T	0.0533	8.76E-06
rs7219031	A	0.0596	2.59E-07
rs72699046	C	0.0897	1.53E-07
rs72889922	A	0.2504	2.91E-06
rs7294375	T	-0.053	3.33E-07
rs7314740	A	-0.0859	7.00E-06
rs7435890	A	0.057	8.71E-08
rs74808730	A	-0.0828	8.69E-06
rs7488386	A	-0.0412	9.15E-06
rs74976226	T	-0.1435	9.66E-06
rs7538546	T	0.0862	6.29E-06
rs7549874	T	0.0495	2.07E-06
rs75630045	A	0.2002	3.29E-06
rs76893867	C	0.0506	8.07E-06
rs77559139	A	0.1371	5.27E-07
rs7917880	A	-0.0427	5.26E-06
rs79280766	A	0.1434	2.20E-06
rs79332111	T	-0.0661	4.85E-06
rs79485249	A	-0.1021	3.11E-07
rs8103309	T	0.0522	3.70E-07
rs879324	A	0.0567	1.90E-06
rs9386182	A	-0.0467	4.22E-06
rs9502286	A	0.1064	6.92E-06
rs9521635	T	-0.0423	9.36E-06
rs9613666	T	0.0426	6.80E-06
rs9668073	T	0.0431	3.97E-06
rs9852938	A	0.0586	9.41E-06
rs9936039	A	-0.0561	1.98E-07
